# Exploring Female Mice Interstrain Differences Relevant for Models of Depression

**DOI:** 10.3389/fnbeh.2015.00335

**Published:** 2015-12-10

**Authors:** Daniela de Sá-Calçada, Susana Roque, Carlos Branco, Susana Monteiro, Bruno Cerqueira-Rodrigues, Nuno Sousa, Joana A. Palha, Margarida Correia-Neves

**Affiliations:** ^1^Life and Health Sciences Research Institute (ICVS), School of Health Sciences, University of MinhoBraga, Portugal; ^2^ICVS/3B's Research Group – PT Government Associate LaboratoryBraga, Portugal

**Keywords:** mouse strains, depressive-like behavior, anxious-like behavior, cytokines, dentate gyrus neurons' morphology, hippocampal cell proliferation, females

## Abstract

Depression is an extremely heterogeneous disorder. Diverse molecular mechanisms have been suggested to underlie its etiology. To understand the molecular mechanisms responsible for this complex disorder, researchers have been using animal models extensively, namely mice from various genetic backgrounds and harboring distinct genetic modifications. The use of numerous mouse models has contributed to enrich our knowledge on depression. However, accumulating data also revealed that the intrinsic characteristics of each mouse strain might influence the experimental outcomes, which may justify some conflicting evidence reported in the literature. To further understand the impact of the genetic background, we performed a multimodal comparative study encompassing the most relevant parameters commonly addressed in depression, in three of the most widely used mouse strains: Balb/c, C57BL/6, and CD-1. Moreover, female mice were selected for this study taken into account the higher prevalence of depression in women and the fewer animal studies using this gender. Our results show that Balb/c mice have a more pronounced anxious-like behavior than CD-1 and C57BL/6 mice, whereas C57BL/6 animals present the strongest depressive-like trait. Furthermore, C57BL/6 mice display the highest rate of proliferating cells and *brain-derived neurotrophic factor (Bdnf)* expression levels in the hippocampus, while hippocampal dentate granular neurons of Balb/c mice show smaller dendritic lengths and fewer ramifications. Of notice, the expression levels of *inducible nitric oxide synthase (iNos)* predict 39.5% of the depressive-like behavior index, which suggests a key role of hippocampal iNOS in depression. Overall, this study reveals important interstrain differences in several behavioral dimensions and molecular and cellular parameters that should be considered when preparing and analyzing experiments addressing depression using mouse models. It further contributes to the literature by revealing the predictive value of hippocampal *iNos* expression levels in depressive-like behavior, irrespectively of the mouse strain.

## Introduction

Depression is a heterogeneous disorder which underlying causes are still poorly understood. Distinct mechanisms have been suggested to be responsible for this disease, the most well studied being dysfunction of the hypothalamic-pituitary-adrenal (HPA) axis (Pariante and Lightman, 2008); decreased neurogenesis, particularly in the hippocampus (Jacobs et al., 2000); and/or alterations in cytokine production leading to a sustained pro-inflammatory profile (Smith, 1991; Schiepers et al., 2005; Miller et al., 2009).

The use of animal models has greatly contributed to the present knowledge on the molecular mechanisms associated with depression. Mice have been particularly relevant, given the availability of so many genetically modified strains that allow the study of specific molecules/pathways. Of notice, it has been shown that the consequences of the altered expression of a specific gene on depressive-like behavior depend on the genetic background of the animals, i.e., on the mouse strain (Gerlai, 1996; Crawley et al., 1997; Yoshikawa et al., 2002; Kastenberger et al., 2012). For instance, deficiency of *Pdyn* (an opioid polypeptide hormone) expression induces opposite anxiety-like phenotypes on Balb/c and C57BL/6 animals (Kastenberger et al., 2012). Moreover, genetic background is also related with distinct responses to specific treatments and procedures (Crawley et al., 1997). For example, four inbred mouse strains showed different locomotion and depressive-like behavior outcomes upon the same fluoxetine's treatment (Dulawa et al., 2004); seven inbred mouse strains were shown to respond differently, in terms of behavior and various biometric and molecular parameters, to an unpredictable chronic mild stress protocol and treatment with imipramine (Ibarguen-Vargas et al., 2008).

Previous studies have also revealed basal specific strain differences with respect to particular behavior phenotypes. Balb/c mice were shown to present a marked anxiety trait (Michalikova et al., 2010; An et al., 2011), whereas C57BL/6 animals were shown to display a high immobility time in the forced swimming test (FST) and in the tail suspension test (TST), suggestive of more pronounced depressive-like behavior (Yoshikawa et al., 2002; Miller et al., 2010). With respect to exploratory and locomotor activities, C57BL/6 mice were reported to be more active and explorers than Balb/c (Fraser et al., 2010; An et al., 2011). Although very useful, and already pointing for relevant behavioral strain differences, most of these studies addressed separately a limited number of behavior traits. The present study intended to broaden the approach, by characterizing behavior domains associated with depression; by addressing molecular and cellular correlates (focusing on the three mostly studied hypothesis on the etiology of depression) and making use of three of the mouse strains most commonly used in behavioral studies (two inbred, Balb/c, C57BL/6, and one outbred, CD-1). We chose to perform this study on females, given the higher prevalence of mood disorders in women and the insufficient number of published studies on female animal models (Palanza, 2001; Cryan and Mombereau, 2004).

## Materials and methods

### Animals

CD-1, C57BL/6, and Balb/c female mice were purchased from Charles River Laboratories (Barcelona, Spain). All mice were housed in groups of 5 per cage, under standard laboratory conditions (12 h light/12 h dark cycle, at 22°C, relative humidity of 55%; food and water *ad libitum*). All animals were behaviorally tested at 4 months of age. Three days after the end of the behavioral tests, animals were weighed and sacrificed by decapitation, by trained certified personnel. Adrenals were dissected and weighed. Spleen and hippocampus, assigned for the quantification of messenger RNA (mRNA) by semi-quantitative real-time polymerase chain reaction (qPCR), were macroscopically dissected and stored at −80°C. For the analysis of hippocampal cell proliferation and dendritic structure, brains were collected from anesthetized animals, transcardially perfused with saline, and lastly sacrificed by decapitation. The estrous cycle (proestrus, estrus, metestrus, and diestrus) of each female was determined, immediately after the performance of each behavioral test (in three consecutive days), by vaginal smear examination for the presence of leukocytes, cornified epithelial and nucleated epithelial cells and their proportions in the smear (Byers et al., 2012). To evaluate the impact of the estrous cycle, the strain and the interaction between these two factors on the behavioral parameters analyzed, data were grouped into proestrus/estrus and metestrus/diestrus stages, since it has been described that the estrogen levels of the grouped stages are very similar (Caligioni, 2009). The two-way ANOVA analyses revealed that estrous cycle did not impact the behavioral parameters assessed (Supplementary Table 1).

All experimental procedures were carried out within the light period of the light/dark cycle and conducted in agreement with National guidelines (Portaria n° 1005/92) and with the European Union Directive 2010/63/EU on animal care and experimentation. This study was approved by the Ethical Committee Board of the Portuguese Veterinary Directorate.

### Behavioral tests

Behavioral tests were performed in three consecutive days, between 9 a.m. and 6 p.m., in the following order: open field test (OFT), FST and TST, to assess anxiety first. All the animals performed all the behavioral tests.

#### Open field test

The OFT was performed to assess locomotor and exploratory activities, as well as anxious-like behavior. Animals were placed in the center of an arena (43.2 × 43.2 cm transparent acrylic walls and white floor) and their position and rearings (vertical activity) monitored and recorded by a three 16-beam infrared system (MedAssociates, VT, USA), during 5 min. The total distance traveled by each animal was used to determine locomotor activity and the number and duration of the rearings to determine exploratory behavior. The percentage of distanced traveled by each animal in the center of the arena (10.8 × 10.8 cm), considered an anxiogenic place due to a bright light falling on the area, was used as an indicative measure of general anxiety (Gould et al., 2009).

#### Forced swimming test

The FST was used to evaluate the ability of mice to cope with a stressful and inescapable situation (behavioral despair). In this test, each animal was placed in a cylinder (17 cm of diameter and 30 cm of height) filled with water (25°C) to a depth so the mouse had no solid support for the rear paws or tail. The activity was recorded for a 6 min period and, subsequently, the last 4 min were scored as mobility or immobility. Additionally, the latency to immobility, which corresponds to the time that each animal takes from the beginning of the test to stop for the first time, was assessed. Mice displaying decreased latency to immobility and longer immobilization periods were considered to display higher behavioral despair, which is a sign of depressive-like behavior (Porsolt et al., 1977). The behavior parameters assessed were score by, at least, two independent researchers, blind to the experimental conditions. The inter-observer reliability was evaluated by the intraclass correlation coefficient (ICC). The ICC for average measures in the FST latency was 0.726 (0.543–0.844); in the FST immobility was 0.891 (0.819–0.937). The graphs present the data from one of the raters.

#### Tail suspension test

The TST addresses, as the FST, depressive-like behavior. Mice were suspended by the tail for 6 min. The activity was recorded and, subsequently, the latency and immobility time were manually scored by at least two independent researchers, blind to the experimental conditions. The ICC for the TST latency was 0.893 (0.766–0.951), in the immobility of the TST was 0.966 (0.927–0.984). The data presented in the graphs are from one of the raters. Decreased latency to immobility and longer immobilization periods were considered traits of depressive-like behavior (Steru et al., 1985).

### Corticosterone measurements

Sera corticosterone levels were measured 3 days after the last behavioral test. Blood was collected from the tip of the tail within the first 2 min after animals were removed from their home cage. Blood collection took place between 9 and 10 a.m., corresponding to the beginning of the light period (the basal time-point of the corticosterone production circadian rhythm). Corticosterone concentration was assessed using a radioimmunoassay (RIA) assay kit (Corticosterone Double Antibody RIA kit, MP Biomedicals, NY, USA), following the manufacturer's guidelines. The detection limit of the assay was 15.4 ng/mL.

### Hippocampal cell proliferation: immunohistochemistry and stereological analysis

To assess cell proliferation in the dentate gyrus (DG) using stereological analysis, brains were embedded in optimum cutting temperature compound and snap-frozen. Serial coronal 20 μm sections were cut in a cryostat, extending over the entire length of the hippocampus. To detect Ki67, a nuclear protein expressed in all phases of the cell cycle except the resting phase G0, a mouse monoclonal anti-Ki67 (Novocastra, UK; 1:100 dilution) was used accordingly with standard procedures. The primary antibody was detected by the Ultravision Detection System (Lab Vision, CA, USA), and the reaction developed with 3,3′-diamino- benzidine substrate (Sigma Aldrich, MO, USA; DAB: 0.025 and 0.15% H_2_O_2_ in Tris-HCl 0.05 M, pH 7.2). Sections were then counterstained with hematoxylin.

Hippocampal cell proliferation was measured by counting the cells expressing Ki-67 in the subgranular zone (SGZ), considered as the 3-cell-body-wide zone at the border of the DG and normalized by the respective area (results are presented as number of Ki67^+^ cells per mm^2^). The use of the visiopharm integrator system software (Visiopharm, Denmark) allowed the delimitation, at low magnification (40x), of the areas of interest and the identification of the Ki67^+^ cells within the defined areas was performed at higher magnification (400x). Counts were performed by one researcher blind to the experimental conditions.

### Dendritic structure

To analyze the dendritic structure, the mouse brains were immersed in Golgi-Cox solution and kept in the dark for 14 days, at room temperature (Glaser and Van der Loos, 1981) and then transferred to a 30% sucrose solution and cut on a vibratome. Coronal sections (200 μm thick) were collected in 6% sucrose and blotted dry onto gelatin-coated microscope slides. They were subsequently alkalinized in 18.7% ammonia, developed in Dektol (Kodak, Rochester, NY, USA), fixed in Kodak Rapid Fix, dehydrated, xylene cleared, mounted, and coverslipped with entellan. All incubation steps were performed in a dark room. To minimize bias, each brain was coded to keep the experimenter blind to the genotype.

The arrangement of dendritic material in the granule cells from the DG of the hippocampus was then analyzed having in consideration the following criteria: (1) full Golgi-impregnation along the dendritic tree; (2) complete dendrites without truncated branches; and (3) relative isolation from neighboring impregnated neurons, astrocytes, or blood vessels to avoid interference with the analysis. Slides containing the region of interest were randomly searched and the first 5–8 neurons fulfilling the criteria (maximum of three neurons per section) were selected.

For each selected neuron, all branches of the dendritic tree were reconstructed, at high magnification (600x), using a motorized microscope (Axioplan 2; Carl Zeiss, Germany) and Neurolucida software (Microbrightfield, VT, USA). A 3D version of a Sholl analysis (Sholl, 1956; Uylings and van Pelt, 2002) of the reconstructed neurons was performed using NeuroExplorer software (Microbrightfield, Inc.; VT, USA); the number of intersections of dendrites with concentric spheres positioned at radial intervals of 10 μm was counted. Additionally, the total length of the dendritic tree was measured. For each group of 5 mice per strain, 30 dentate granule cells were analyzed.

### mRNA expression levels by qPCR

The mRNA levels of the genes encoding for *interferon* (*Ifn)-*γ, *interleukin* (*Il)-1ß, tumor necrosis factor* (*Tnf), Il-10*, and *inducible nitric oxide synthase* (*iNos)* were measured by qPCR in the spleen and in the hippocampus. The expression levels of the *brain-derived neurotrophic factor* (*Bdnf*) were assessed in the hippocampus.

Total RNA (1 μg) was reverse transcribed using iScript cDNA synthesis kit (Bio-Rad, Hercules, CA, USA). The geometric mean of the mRNA expression levels of three different genes were used as reference: *hypoxanthine guanine phosphoribosyl transferase* (*Hprt*); *glyceraldehyde 3-phosphate dehydrogenase* (*Gapdh*) and; *18S ribosomal RNA* (*18S*) (Vandesompele et al., 2002). qPCR reactions were performed on a CFX96 Real-Time PCR Detection System (Bio-Rad, CA, USA) using EVA Green (Bio-Rad, CA, USA).

### Data analyses

Normal distribution of the variables was tested using the Shapiro-Wilk test (normal distribution was confirmed for all the variable assessed; *p* > 0.05). One-way ANOVA was applied to all the data, except for the results from the Sholl analysis of the reconstructed neurons in which ANOVA repeated-measures was applied using the mean obtained for each animal. The differences between the groups were analyzed by the Tukey *post-hoc* honestly significant difference test for α = 0.05. The effect size was evaluated by the eta square (η^2^), dividing the between-groups sum of squares by the total sum of squares and considering η^2^ ≥ 0.01 a small; ≥ 0.06 a medium; and ≥ 0.14 a large effect (Cohen, 1988).

To correlate the behavioral phenotype with the distinct parameters analyzed, the Pearson correlation coefficient test was used.

To calculate a depressive-like behavior index, in accordance with the previously described emotionality score (Guilloux et al., 2011), we transformed FST and TST latency and immobility times into Z-scores (to achieve normalized mean values for each behavioral test), calculated the symmetric values of the latency Z-scores and performed an average of those four variables for each animal. A linear regression was conducted to test the ability to predict the value for the depressive-like index, using *iNos* expression levels in the spleen and hippocampus as potential predictors (the variables that present statistical significant correlations with the parameters used to assess depressive-like behavior).

The results shown are expressed as mean + SEM and correspond to one of two independent experiments with similar results. All data were analyzed with the GraphPad Prism software (v.6, GraphPad software Inc. CA, USA), apart from the correlation and regression analyses that were performed with the SPSS statistics software (v.22, SPSS Inc. IL, USA).

## Results

### CD-1, C57BL/6, and Balb/c mice display distinct locomotor, anxious, and depressive-like behavioral traits

**Figure 1:**
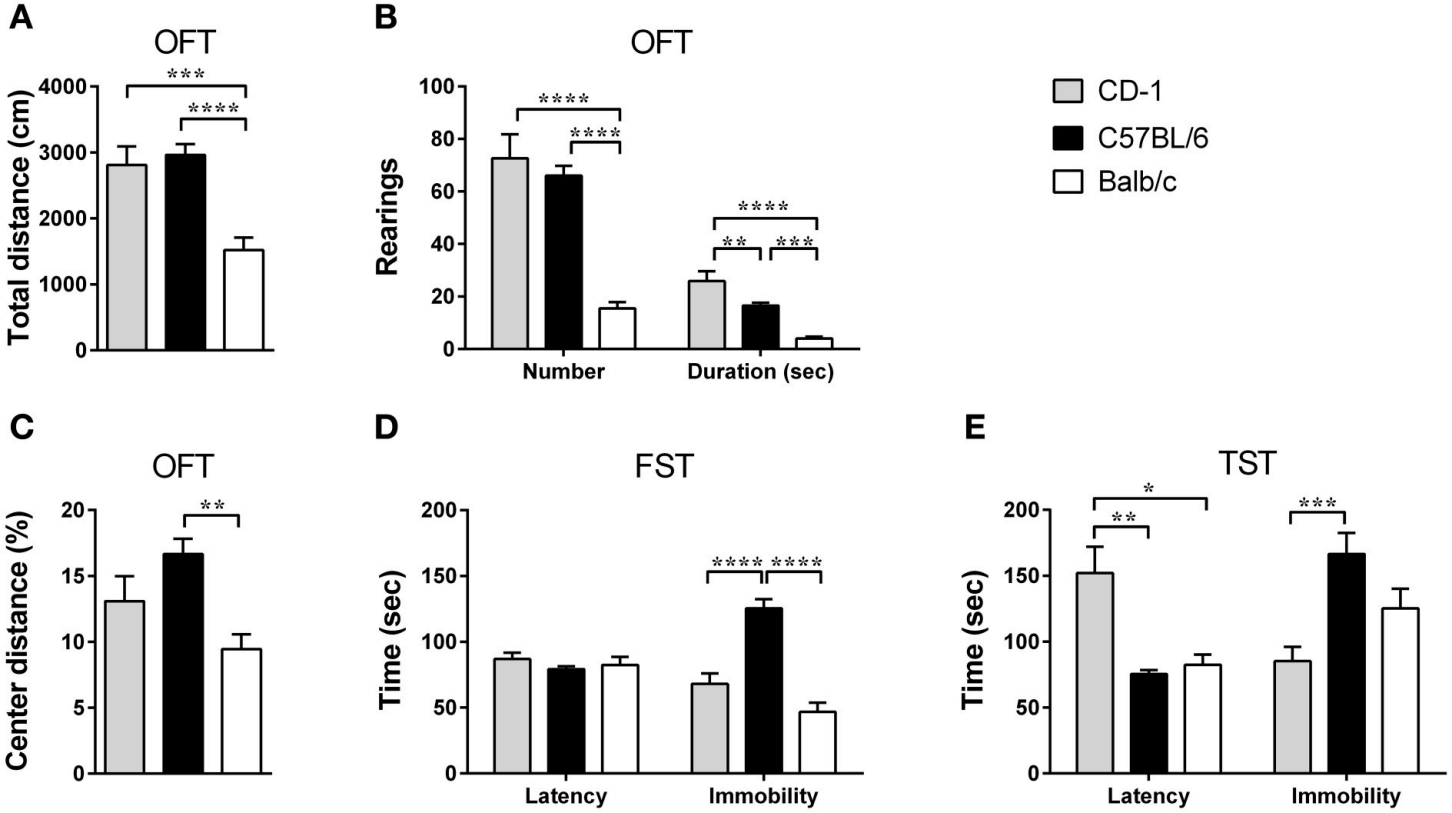
**The three mouse strains present differences in locomotor and exploratory activities, as well as in anxious and depressive-like behaviors**. OFT, FST, and TST were performed in CD-1 (gray bars), C57BL/6 (black bars), and Balb/c (white bars) mice. In the OFT the total distance traveled **(A)**, the number and duration of rearings **(B)**, and the percentage of distance traveled in the center of the OFT's arena **(C)** were scored, and used as a measure of locomotion, exploratory activity, and anxious-like behavior, respectively. In the FST **(D)** and TST **(E)**, the latency to immobility and immobility times were scored and used as measures of depressive-like behavior. Each bar represents the mean + SEM from 11 to 21 mice of 1 of 2 independent experiments with similar results. ^*^p < 0.05; ^**^p < 0.01; ^***^p < 0.001; ^****^p < 0.0001.

The OFT revealed that the mouse genetic background significantly impacts locomotor [Figure 1A; *F*_(2, 48)_ = 14.16, *p* < 0.0001, η^2^ = 0.37] and exploratory activities [Figure 1B; number: *F*_(2, 48)_ = 31.0; *p* < 0.0001; η^2^ = 0.56; duration: *F*_(2, 48)_ = 26.26; *p* < 0.0001; η^2^ = 0.52] with Balb/c mice displaying an overall decreased activity. Mice from the Balb/c strain traveled shorter distances in the OFT in comparison to CD-1 (*p* = 0.0004) and C57BL/6 mice (*p* < 0.0001) and performed less and shorter rearings, in comparison to CD-1 (p_number_ < 0.0001; p_duration_ < 0.0001) and C57BL/6 mice (p_number_ < 0.0001; p_duration_ = 0.0002). Additionally, C57BL/6 mice showed lower rearing duration than CD-1 mice (p_duration_ = 0.0061).

For the anxious-like behavior, tested using the ratio of distance travaled in the center vs. total distance of the OFT [Figure 1C; *F*_(2, 47)_ = 7.06, *p* = 0.0021, η^2^ = 0.23], Balb/c mice showed a more pronounced anxious-like behavior by displaying a lower percentage of distance traveled in the center of the OFT's arena, in comparison to C57BL/6 mice (*p* = 0.0014).

Depressive-like behavior was addressed using the FST and the TST. When immobility was analyzed, both tests revealed differences between strains [FST: Figure 1D; *F*_(2, 42)_ = 30.55; *p* < 0.0001; η^2^ = 0.59; TST: Figure 1E; *F*_(2, 33)_ = 9.47; *p* = 0.0006; η^2^ = 0.36], with C57BL/6 mice showing a higher immobility time in comparison to CD-1 in both tests (FST: *p* < 0.0001; TST: *p* = 0.0004) and Balb/c mice in the FST (*p* < 0.0001). On the other hand, the analysis of the latency to immobility time showed interstrain differences only in the TST [Figure 1E; *F*_(2, 31)_ = 7.86; *p* = 0.0017; η^2^ = 0.34], with CD-1 mice displaying a longer latency time than C57BL/6 (*p* = 0.0040) and Balb/c mice (*p* = 0.0118) (Figure 1E).

### CD-1, C57BL/6, and Balb/c mice do not present differences on corticosterone serum levels

**Figure 2:**
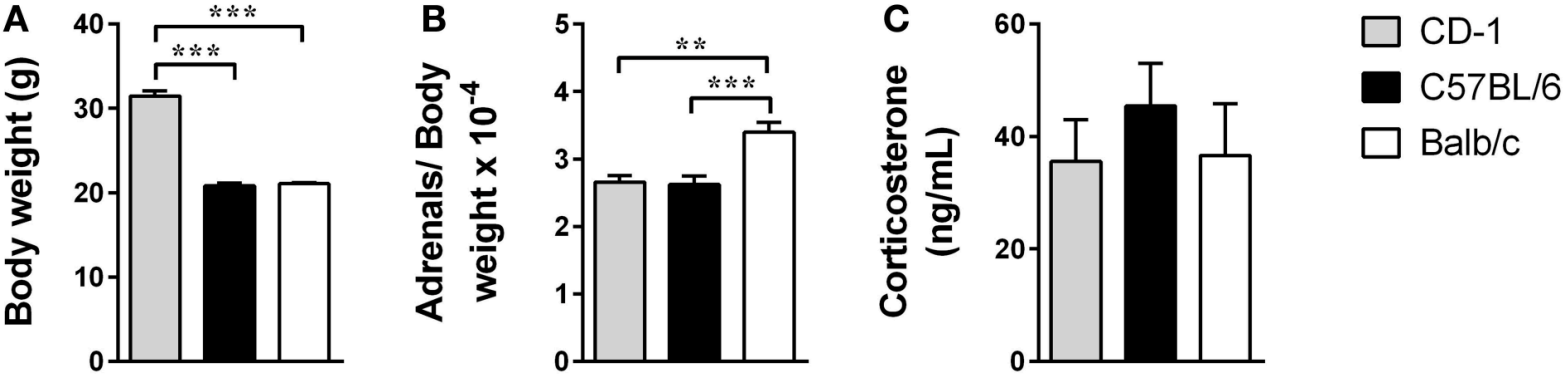
**The three mouse strains do not reveal significant differences in the corticosterone serum levels**. Body weight **(A)**, adrenals weight (normalized to the body weight) **(B)**, and serum corticosterone basal levels **(C)** were measured in CD-1 (gray bars), C57BL/6 (black bars), and Balb/c (white bars) mice. Each bar represents the mean + SEM from 9 to 16 mice of 1 of 2 independent experiments. ^**^p < 0.01; ^***^p < 0.001.

Considering that a putative dysfunction of the HPA axis, and the consequent increased glucocorticoids levels, has been widely investigated as a potential mechanism involved in depression's etiology (Pariante and Lightman, 2008) we assessed the weight of the adrenal glands and the corticosterone basal levels. To determine the adrenals weight normalized to the mouse body weight we first determined the weight of the mice. There were clear differences in the body weight of the three strains [Figure 2A; *F*_(2, 26)_ = 197.5; *p* < 0.0001; η^2^ = 0.94], with animals from the CD-1 genetic background being heavier than C57BL/6 (*p* < 0.0001) and Balb/c mice (*p* < 0.0001). The ratio adrenals/body weight revealed significant differences between the three strains [Figure 2B; *F*_(2, 26)_ = 11.44; *p* = 0.0003; η^2^ = 0.47], namely between Balb/c and CD-1 (*p* = 0.0011) and C57BL/6 (*p* = 0.0006). However, serum corticosterone basal levels were not significantly different between the three strains [Figure 2C; *F*_(2, 40)_ = 0.4595; *p* = 0.6349].

### CD-1, C57BL/6, and Balb/c mice present differences in hippocampal cell proliferation and dendritic morphology

Taking into account the interstrain differences observed in anxious and depressive-like behaviors and studies that propose alterations in cell proliferation, *Bdnf* expression and neuronal morphology in the hippocampus as playing a role in the etiology of depression (Jacobs et al., 2000; Groves, 2007; Bessa et al., 2009), we next analyzed these parameters.

**Figure 3:**
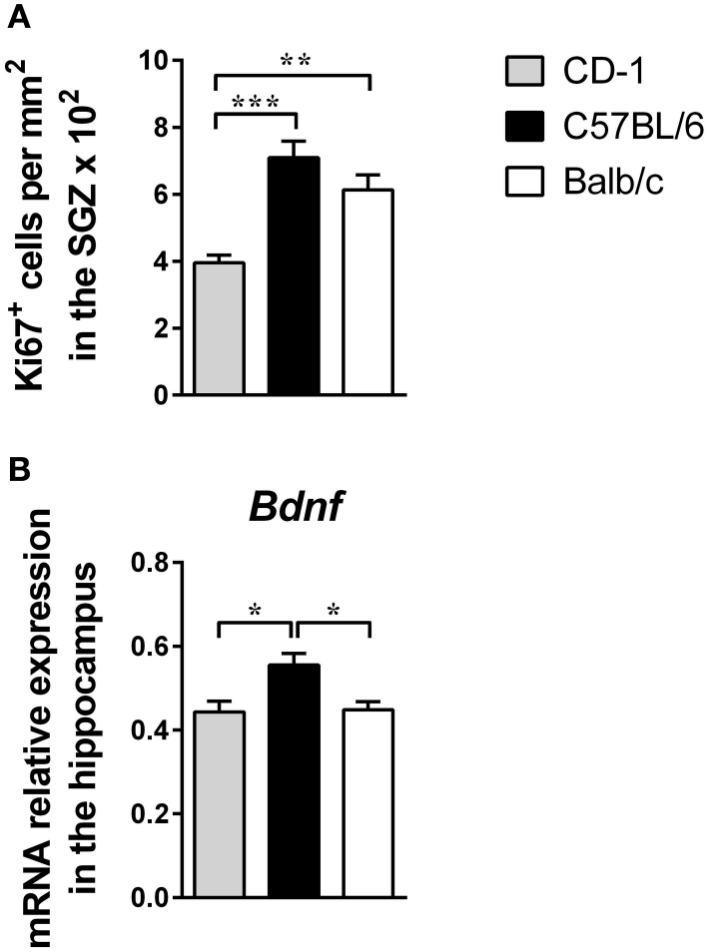
**The three mouse strains display significant differences in cell proliferation and *Bdnf* mRNA level expression in the hippocampus**. Hippocampal cell proliferation **(A)** and the *Bdnf* expression levels in the hippocampus **(B)** were measured in CD-1 (gray bars), C57BL/6 (black bars), and Balb/c (white bars) mice. Hippocampal cell proliferation data **(A)** are presented as mean + SEM of Ki67^+^ cells per mm^2^ in the SGZ of each animal (6–8 mice per strain from the 2 independent experiments). The mRNA expression level **(B)** was normalized using three reference genes *Hprt, Gapdh, 18S* and each bar represents the mean + SEM from 8 mice of 1 of 2 independent experiments with similar results. ^*^p < 0.05; ^**^p < 0.01; ^***^p < 0.001.

The assessment of hippocampal cell proliferation revealed interstrain differences [Figure 3A; *F*_(2, 18)_ = 14.97; *p* = 0.0001; η^2^ = 0.62], with CD-1 mice presenting a lower number of Ki-67^+^ cells in the DG, compared to C57BL/6 (*p* = 0.0002) and Balb/c mice (*p* = 0.0027). The levels of *Bdnf* mRNA in the hippocampus were also significantly different between the three strains [Figure 3B; *F*_(2, 21)_ = 6.530; *p* = 0.0062; η^2^ = 0.38], with C57BL/6 mice displaying higher levels in comparison to CD-1 (*p* = 0.0114) and Balb/c mice (*p* = 0.0160).

**Figure 4:**
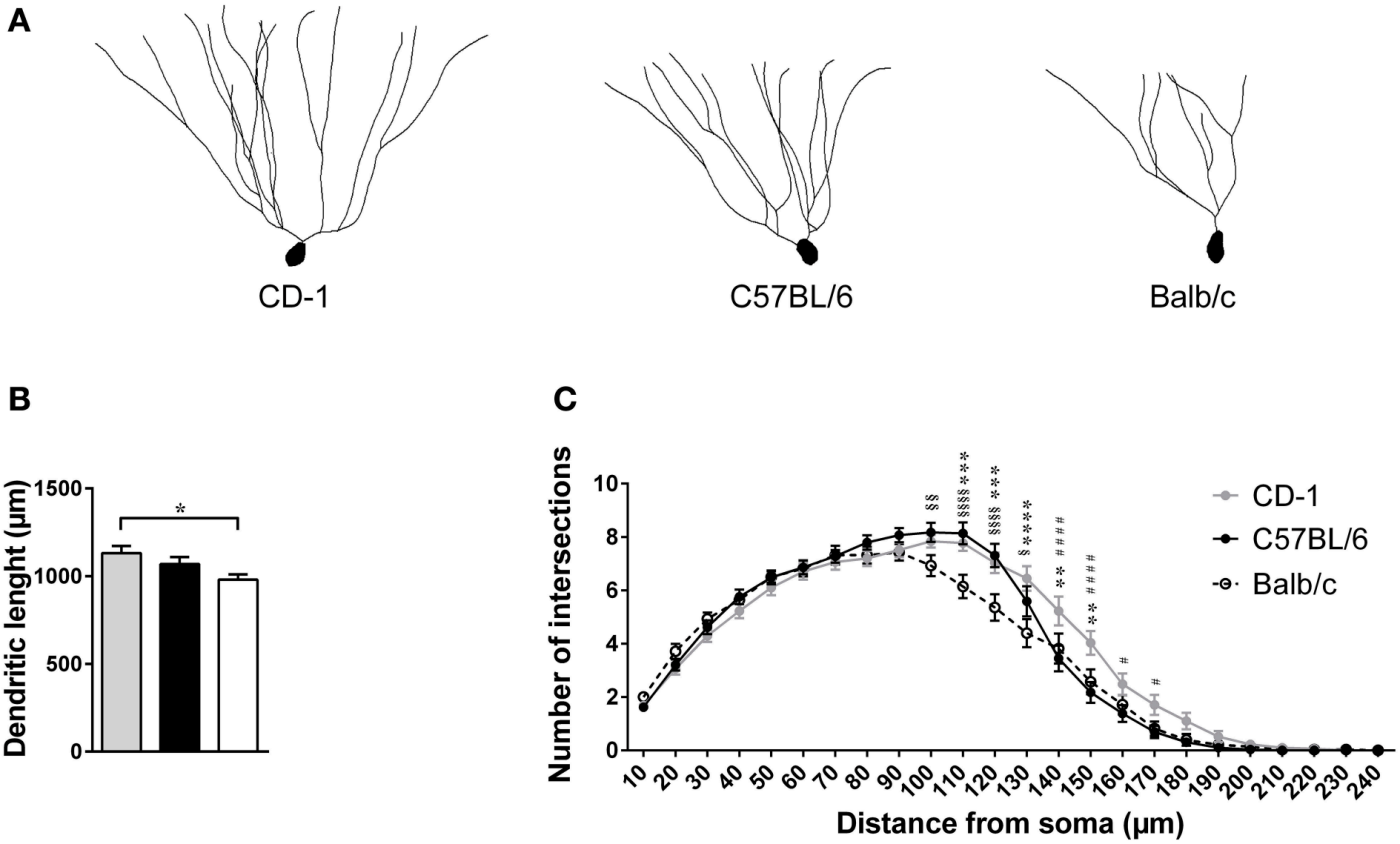
**The three mouse strains present significant morphological differences in their granule neurons**. Dendritic morphology of granule neurons from the dentate gyrus (DG) was analyzed in CD-1 (gray bar, symbol, and line), C57BL/6 (black bar, symbol, and line), and Balb/c (white bar, unfiled symbol, and dashed line) mice. Representative dentate granule neurons of the hippocampus of each mouse strain are shown **(A)**. The total length of the dendritic tree **(B)** and Sholl analysis of the number of intersections of dendrites at specific distances from the soma are displayed **(C)**. Each bar represents the mean + SEM of the average length of 28–30 granule neurons per group, from 5 mice per strain; the lines represent the average number of intersections of dendrite branches with consecutive 10 μm-spaced concentric spheres of the same neurons. ^*^Balb vs. CD1; ^§^Balb vs. B6; #CD1 vs. B6; 1 symbol *p* < 0.05; 2 symbols *p* < 0.01; 3 symbols *p* < 0.001; 4 symbols *p* < 0.0001.

The morphological analysis of DG's granule neurons revealed significant differences in the total length of the dendrites [Figures 4A,B; *F*_(2, 82)_ = 3.901; *p* = 0.0241; η^2^ = 8.69 × 10^−4^], with neurons from Balb/c mice presenting shorter dendritic trees in comparison to those from CD-1 (*p* = 0.0178). The arrangement of the dendritic material of these same neurons, assessed by the number of intersections of dendrites as a function of their distance from the soma, did not reveal differences between the mouse strains [Figure 4C; *F*_(2, 85)_ = 1.723; *p* = 0.1876]. However, the *post-hoc* analyses revealed that mice from Balb/c strain present less dendritic ramifications (less intersections) at distances around 100–130 μm from the soma, relatively to CD-1 and C57BL/6 mice. At distances greater than 130 μm from the soma, the granule neurons from CD-1 mice presented higher dendritic ramifications (more numbers of intersections) in comparison to the other two mouse strains.

### CD-1, C57BL/6, and Balb/c mice present different cytokine profiles in the periphery and in the hippocampus

**Figure 5:**
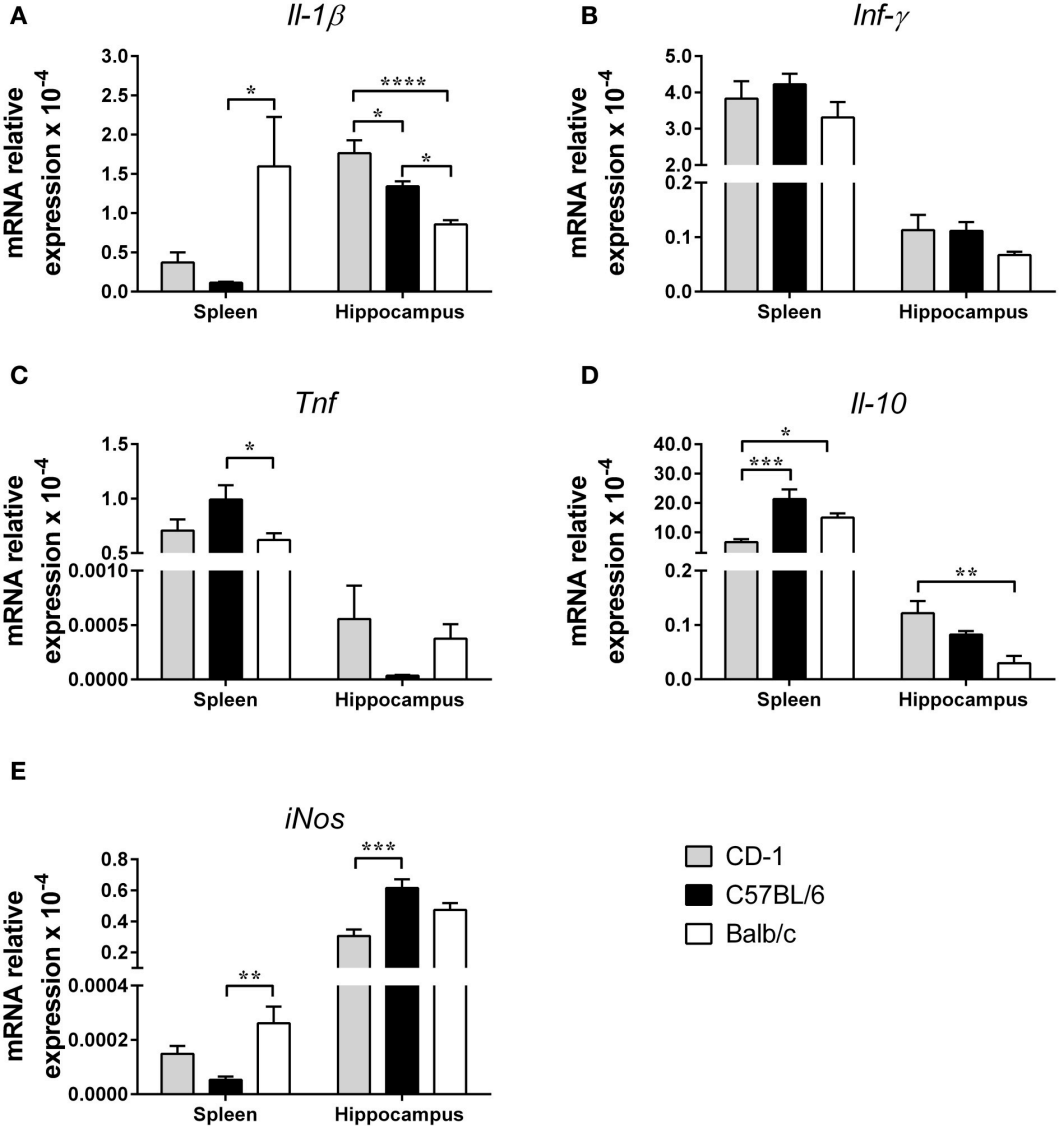
**The three mouse strains present distinct cytokine profiles in the spleen and in the hippocampus**. mRNA expression for the pro-inflammatory cytokines *Il-1*β **(A)**, *Ifn-*γ **(B)***, Tnf* **(C)**, the anti-inflammatory cytokine *Il-10* **(D)**, and the enzyme *iNos* **(E)** were measured in the spleen and in the hippocampus of CD-1 (gray bars), C57BL/6 (black bars), and Balb/c (white bars) mice. The mRNA expression levels were normalized using three reference genes *Hprt, Gapdh*, and *18S*. Each bar represents the mean + SEM from 6 to 8 mice per strain of 1 of 2 independent experiments with similar results. ^*^p < 0.05; ^**^p < 0.01; ^***^p < 0.001; ^****^p < 0.0001.

Alterations in the cytokine profile have been associated with mood disorders (Smith, 1991; Schiepers et al., 2005; Miller et al., 2009) and with potential mechanisms underlying those behavioral phenotypes (Haroon et al., 2012). Thus, we next analyzed the expression levels of the genes encoding for three of the most relevant pro-inflammatory cytokines, IFN-**γ**, IL-1β, and TNF, for the anti-inflammatory cytokine IL-10 and for the enzyme iNOS, all previously addressed in studies on the etiology of depression. This profiling was investigated in the hippocampus, a brain region known to be involved in depression and anxiety, and in the spleen, one of the most important organs of the immune system. The data presented in Figure 5 shows that strains display a distinct cytokine profile and that, within each strain, there are organ differences. A striking example is the organ differential expression of *Il-1*β [Figure 5A spleen: *F*_(2, 21)_ = 4.587; *p* = 0.0022; η^2^ = 0.30; hippocampus: *F*_(2, 20)_ = 16.19; *p* < 0.0001; η^2^ = 0.62]; of interest Balb/c is the strain showing higher expression in the spleen (*p* = 0.0258 comparatively to C57BL/6) and lower expression in the hippocampus (Balb/c vs. CD-1: *p* < 0.0001; Balb/c vs. C57BL/6: *p* = 0.0167). No differences were detected in the mRNA expression level of *Ifn-*γ in the spleen [Figure 5B; *F*_(2, 20)_ = 1.228; *p* = 0.3141] and hippocampus [Figure 5B; *F*_(2, 20)_ = 1.641; *p* = 0.2189]. Regarding *Tnf* expression, differences between the three strains were only detected in the spleen [Figure 5C; *F*_(2, 21)_ = 3.553; *p* = 0.0469; η^2^ = 0.25], namely between C57BL/6 and Balb/c mice (*p* = 0.0474). The pattern of *Il-10* mRNA expression, like the one observed for *Il-1*β, revealed differences between strains and between organs within the same strain [Figure 5D; spleen: *F*_(2, 20)_ = 10.59; *p* = 0.007; η^2^ = 0.52; hippocampus: *F*_(2, 13)_ = 7.117; *p* = 0.0082; η^2^ = 0.52]; while C57BL/6 is the strain with higher expression in the spleen (*p* = 0.0004, when compared with CD-1); CD-1 mice displayed higher expression in the hippocampus (*p* = 0.0061, when compared with Balb/c). Lastly, the results for *iNos* mRNA expression revealed differences in the spleen [Figure 5E; *F*_(2, 21)_ = 6.777; *p* = 0.0054; η^2^ = 0.39] between C57BL/6 and Balb/c mice (*p* = 0.0038), and in the hippocampus [Figure 5E; *F*_(2, 21)_ = 10.63; *p* = 0.0006; η^2^ = 0.50] between CD-1 and C57BL/6 mice (*p* = 0.0004).

### *iNos* expression predicts the depressive-like behavior index

**Figure 6:**
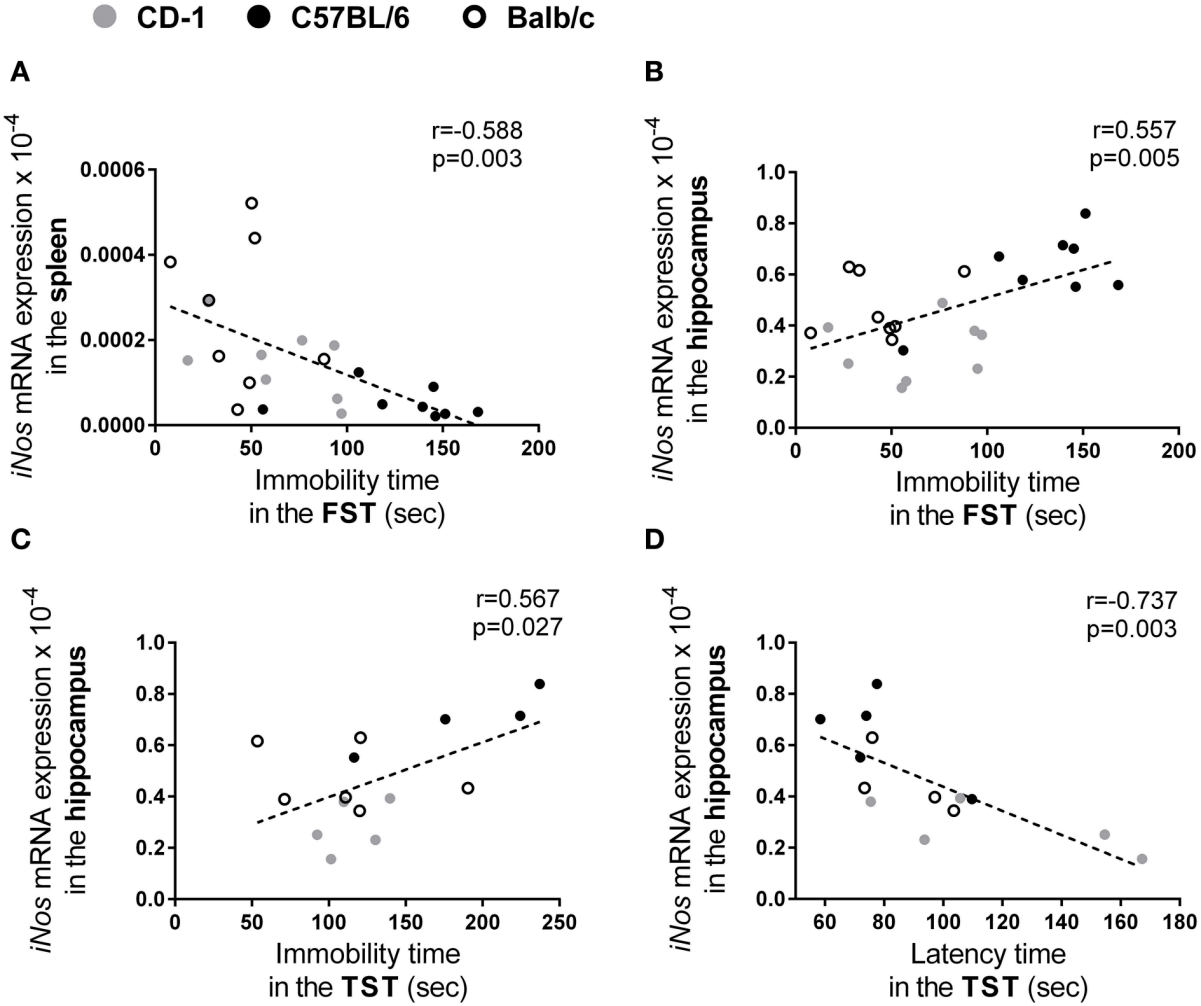
**Independently of the mouse strain, the *iNos* expression level is correlated with several parameters used to assess depressive-like behavior**. From the parameters assessed in CD-1 (gray symbols), C57BL/6 (black symbol), and Balb/c (hollow symbol) mouse strains, statistically significant correlations were found between the mRNA expression levels of *iNos* in the spleen and the immobility time in the FST **(A)**; the mRNA expression levels of *iNos* in the hippocampus and the immobility time in the FST **(B)**, immobility **(C)**, and latency times in the TST **(D)**. Each symbol represents one animal.

In the present study, in order to identify and measure the association between the different parameters assessed in the three mouse strains, we performed a correlation analysis between the behavior and the molecular/cellular parameters analyzed. We found correlations between the levels of *iNos* mRNA expression and parameters of depressive-like behavior: in the spleen the expression level of this enzyme was found to be negatively correlated to immobility time on the FST (Figure 6A; *r* = −0.588; *p* = 0.003); whereas in the hippocampus was found to be positively correlated to the immobility time in the FST (Figure 6B; *r* = 0.557; *p* = 0.005) and in the TST (Figure 6C; *r* = 0.567; *p* = 0.027) and negatively correlated to the latency time in the TST (Figure 6D; *r* = −0.737; *p* = 0.003).

Taken into account the correlations observed between the *iNos* expression and the several parameters that assess depressive-like behavior, we used a linear regression model to test their predictive value (Table 1). Since depression was investigated using two different tests we first calculated a depressive-like index (an average of the latency and immobility times transformed into Z-scores from the FST and TST). A significant regression model emerged revealing that the expression levels of *iNos* in the spleen and in the hippocampus account for 39.5% of the variance in the depressive-like index [*F*_(2, 10)_ = 4.916, *p* = 0.033]; the expression levels of *iNos* in the hippocampus was the only parameter that revealed to be a statistical significant predictor (*p* = 0.025).

**Table 1:** **Regression analysis to predict the depressive-like behavior index**.

**Regression analysis predicting**	**Variables (mRNA expression levels)**	***R*^2^**	***R*^2^ adj**	***F***	**df**	**β**	***t***
Depressive-like index	*iNos* hippocampus	0.496	0.395	4.916^*^	2.10	0.620	2.624^*^
	*iNos* spleen					−0.192	−0.812

Inducible nitric oxide synthase (iNos);

* *p < 0.05*.

## Discussion

In this work we performed a multimodal assessment of the three most widely used mouse strains (CD-1, C57BL/6, and Balb/c) and analyzed behavioral, cellular, and molecular parameters that have been associated with the etiology of depression. The findings reported here are relevant in two dimensions: (i) they confirm, strengthen and render far more comprehensive previous scattered results on a set of parameters used to investigate depressive-like behavior for the three mouse strains; (ii) by combining a set of behavioral and molecular/cellular parameters, this study adds to the literature novel correlations that might contribute for the definition of new strategies to treat depression.

The analyses of the behavioral phenotype revealed that, from the three genetic backgrounds used, female Balb/c mice are the less active and explorative, as it was previously shown by studies performed in both genders (Lalonde and Strazielle, 2008) or just in females (An et al., 2011). These observations are also in accordance to other studies showing that, among the inbred strains, the C57 mouse strains (C57 is used as a general abbreviation referring to several inbred black mouse strains including C57BL/6) consistently show high levels of locomotion, whereas Balb/c mice typically exhibit low locomotor activity (Crawley et al., 1997; Fraser et al., 2010; Michalikova et al., 2010). Additionally, the present OFT's results indicate that Balb/c mice have a more pronounced anxious-like behavior in comparison to C57BL/6. Crawley and collaborators had already shown that Balb/c mice typically exhibit high levels of emotional reactivity, whereas C57 mice tend to display low levels of anxiety-related measures in the OFT (Crawley et al., 1997). Of relevance, one other study using males from the same three mouse strains observed analogous results to those we found here in females, although using a different apparatus (Michalikova et al., 2010). To assess depressive-like behavior we used two different paradigms: the FST (Porsolt et al., 1977) and the TST (Steru et al., 1985). In both tests, female C57BL/6 mice revealed longer immobility periods, as previously reported by other authors using both females and males (Lucki et al., 2001; Yoshikawa et al., 2002). These results suggest a more pronounced despair/depressive-like behavior in female C57BL/6 mice comparatively to the other strains analyzed, which do not seem to be associated with locomotion since, in the OFT, the strain displaying lower locomotor activity is the Balb/c. The CD1 mice present an increased latency to immobility in the TST suggesting a decreased depressive-like behavior, which is in accordance with the immobility time observed both in TST and FST when compared with the C57BL/6 mice. However, this behavioral phenotype is not corroborated by the latency to immobility in the FST. Although FST and TST are two well validated paradigms to evaluate depressive-like behavior, differences in the performance of those tests have been described, suggesting that they could evaluate slightly different pathophysiological mechanisms (Yoshikawa et al., 2002; Castagné et al., 2010). Still, most of the results are very consistent in both behavioral tests. We were interested in further correlating morphological and molecular aspects of depression with behavioral performance, centered in three of the most studied theories of depression. With respect to the HPA axis hypothesis, we observed heavier adrenals/body weight in Balb/c in comparison to the other two mouse strains. These results are consistent with those reported by Deschepper and collaborators who showed that Balb/c animals display higher adrenals weight than C57BL/6 mice (Deschepper et al., 2004). While the lack of correspondence between the adrenals/body weight ratio and the sera corticosterone levels found between the three mouse strains could be somehow unexpected, this is actually in agreement with one study reporting similar basal corticosterone levels in these strains of mice (Shanks et al., 1990). In fact, it is presently accepted that no major interstrain differences exist in basal corticosterone sera levels (Shanks et al., 1990; Anisman et al., 1998).

The results obtained on the hippocampal cell proliferation in the DG showed that CD-1 mice present a lower proliferating rate in comparison to C57BL/6 and Balb/c. A similar pattern between the three mouse strains was also observed by other authors, even though no statistical significant differences have been previously reported between CD-1 and Balb/c strains (Kempermann et al., 1997). This small discrepancy may be due to differences in the technical approach used to quantify the proliferating cells: while we used the Ki67 marker, that is expressed in all phases of the cell cycle except the resting phase G0, Kempermann and collaborators labeled the dividing cells using BrdU which is incorporated into the DNA during the S-phase of the mitotic process (Kee et al., 2002). The strain with the highest number of Ki67^+^ cells, the C57BL/6 mice, presented the highest *Bdnf* expression levels, which is in agreement with BDNF signaling playing a role in the maintenance of the basal rate of neural stem cell proliferation (Lee et al., 2002). Interestingly, the C57BL/6 strain, the one that presents higher hippocampal cell proliferation, also shows more pronounced depressive-like behavior. These observations seem to be somehow in contrast with what has been shown in respect to hippocampal cell proliferation in animal models of depression. In fact, depression has been associated with decreased adult hippocampal neurogenesis and interventions that increase neurogenesis in animal models of depression revealed beneficial effects on mood (Jacobs et al., 2000; Sahay and Hen, 2007). However, neurogenesis alteration in otherwise healthy animals does not trigger depressive-like behavior (Bessa et al., 2009; Sahay et al., 2011). In fact, rats treated during 2 weeks with methylazoxymethanol, a cytostatic drug that reduces neurogenesis, did not present alterations in sucrose preference neither in the FST (Bessa et al., 2009). Similarly, no alterations in depressive and anxious-like behaviors were observed in a mouse model with increased neurogenesis (caused by an inducible genetic expansion of the adult-born neurons population by enhancing their survival) (Sahay et al., 2011). Overall, these observations suggest that alterations in neurogenesis are not, *per se*, sufficient to induce mood alterations but are crucial in animal models of depression and of antidepressant therapy. Morphological analyses revealed that female Balb/c's dentate granule neurons have shorter dendrites and that their dendritic trees are less ramified, when compared to CD-1 and C57BL/6 mice. Interestingly, Balb/c mice present a more pronounced anxious-like behavior. In fact, hippocampal neurons are integrated in neuronal networks implicated in emotional behavior and thereby can modulate anxiety (Gorman and Docherty, 2010). To our knowledge, no study ever compared the dendritic structure of granule neurons between these mouse strains. Similarly, this is the first study that evaluates the cytokine profile on these mouse strains, in basal physiological conditions. Interestingly, our results reveal that the pattern of cytokine expression in the spleen is very distinct from that in the hippocampus, supporting the idea that the cytokine profile in peripheral immune cells cannot be considered a mirror of what happens in the brain (You et al., 2011). Moreover, the three mouse strains have distinct profile of cytokine expression in the two tissues. We observed that in the hippocampus Balb/c mice present lower levels of *Il-1*β and *Il-10* expression, whereas no interstrain differences were observed in the expression levels of *Tnf* and *Ifn-*γ. On the other hand, C57BL/6 mice present lower expression levels of *iNos* in the spleen but higher in the hippocampus.

Remarkably, the analysis of the three strains together revealed that, from all the genes studied, the *iNos* expression is the only that presents correlation with behavioral parameters associated with depression. Moreover, using a linear regression analysis we found that the expression levels of this enzyme in the spleen and hippocampus accounts for 39.5% of the variance of the depressive-like index. These results are extremely interesting since several studies have already associated this NOS isoform to depressive-like behavior (Wang et al., 2008; Montezuma et al., 2012; Peng et al., 2012), suggesting a deleterious effect of iNOS in depression. iNOS inhibition has been shown to suppress the depressive-like behavior changes induced by a protocol of chronic stress (Wang et al., 2008). In addition, the systemic absence of iNOS (either by selective inhibition or by gene expression ablation) has been shown to induce an antidepressant-like effect (Montezuma et al., 2012) further suggesting that iNOS levels may modulate depressive-like behavior in mice. Interestingly, we observed a significant correlation between hippocampal *iNos* expression and three of the behavioral parameters used to assess depressive-like behavior. This finding is further supported by the linear regression model in which hippocampal *iNos* expression level is the only statistical significant predictor of the depressive-like behavior index. However, a negative correlation was found between immobility in the FST and the spleen expression level of *iNos*. This result, in apparent contradiction with the literature on full iNOS null mice and also with our data on hippocampal *iNos* expression, suggests that only the iNOS in the CNS (and not the one from the periphery) is involved in the behavioral alterations observed. Indeed, specific inhibition of iNOS in the hippocampus, is sufficient to produce antidepressant effects (Montezuma et al., 2012). The possibility that *iNos* expression has implications in the pathophysiology of depression is also supported by studies showing that nitric oxide is an important signaling molecule known to modulate norepinephrine, serotonin, dopamine and glutamate, major neurotransmitters implicated in the neurobiology of depression (Dhir and Kulkarni, 2011). It is important to emphasize the present study was performed in female mice in basal conditions and not after exposure to any stressful stimulus.

A final note on the gender chosen for this study. We choose females because women present higher prevalence rates of depression than men, and because the vast majority of animal studies related to these mood disorder are still carried out in male mice (Palanza, 2001; Cryan and Mombereau, 2004), potentially originating a strong sex bias in basic science's studies (Clayton and Collins, 2014). Of notice, the interstrain differences observed in the females are very similar to the ones formerly reported in males, mainly in terms of behavior, and the estrous cycle did not influence on the behavioral parameters analyzed.

In summary, these results provide evidence for the existence of intrinsic differences among mouse strains in behavioral dimensions and molecular/cellular parameters associated with depression, emphasizing that care must be taken when choosing the mouse strain for behavioral studies. The common findings in the three strains, as is the case for *iNos* expression, strongly support a consistent association of iNOS with depression, which renders it a candidate target in new therapeutic approaches for mood disorders as suggested by other authors (Wang et al., 2008; Montezuma et al., 2012; Peng et al., 2012).

## Author contributions

DS-C, SR, CB, SM, and BC-R performed the behavior assessment and analysis, blood and tissue collection. Both DS-C and SR carried out the corticosterone measurements, estrous cycle analysis and the statistical analysis of the data. DS-C and SM assessed the dendritic structure of the neurons. DS-C assessed the hippocampal cell proliferation and SR the mRNA expression by qPCR. All authors contributed to the planning of the experiments, data interpretation, writing and critical review of the article and approved the final version of the manuscript.

## Funding

This work was funded by the Portuguese North Regional Operational Program (ON.2—O Novo Norte) under the National Strategic Reference Framework (QREN), through the European Regional Development Fund (FEDER). The funders had no involvement into the analysis and interpretation of data, writing of the manuscript or the decision to submit the article for publication.

### Conflict of interest statement

The authors declare that the research was conducted in the absence of any commercial or financial relationships that could be construed as a potential conflict of interest.
