# Supplementary material for: Exploring Female Mice Interstrain Differences Relevant for Models of Depression
Source: Front Behav Neurosci. 2015 Dec 10;9:335. doi: 10.3389/fnbeh.2015.00335 (PMC4674561; doi:10.3389/fnbeh.2015.00335)
Supplement: Supplementary file 1 [file Table1.DOCX]

**Supplementary data**:

**Supplementary table 1**. Results from the impact of the estrous cycle variable in behavior tests using two-way ANOVA test

| **Impact of the variable estrous cycle in:** | | **F (df)** | **p** |
| --- | --- | --- | --- |
| **OF** | Total distance (cm) | 1.737 (1,40) | 0.317 |
|  | Center distance (%) | 3.964 (1,39) | 0.174 |
|  | Number of rearings | 2.076 (1,40) | 0.284 |
|  | Duration of rearings (sec) | 1.599 (1,40) | 0.442 |
| **FST** | Latency (sec) | 0.376 (1,40) | 0.600 |
|  | Immobility (sec) | 3.038 (1,40) | 0.144 |
| **TST** | Latency (sec) | 1.823 (1,38) | 0.269 |
|  | Immobility (sec) | 0.386 (1,40) | 0.141 |

Estrous cycle were used as a random variable
